# The regulation of NLRP3 inflammasome expression during the development of cardiac contractile dysfunction in chronic kidney disease

**DOI:** 10.18632/oncotarget.22964

**Published:** 2017-12-06

**Authors:** Li-Han Chin, Yu-Juei Hsu, Shih-Che Hsu, Yen-Hui Chen, Yung-Lung Chang, Shih-Ming Huang, Chien-Sung Tsai, Chih-Yuan Lin

**Affiliations:** ^1^ Department of Surgery, Tri-Service General Hospital, National Defense Medical Center, Taipei, Taiwan; ^2^ Department of Medicine, Tri-Service General Hospital, National Defense Medical Center, Taipei, Taiwan; ^3^ Department of Biochemistry, National Defense Medical Center, Taipei, Taiwan; ^4^ Institute of Biomedical Sciences, Academia Sinica, Taipei, Taiwan; ^5^ Superintendent’s Office, Taoyuan Armed Forces General Hospital, Taoyuan, Taiwan

**Keywords:** cardiac contractile dysfunction, chronic kidney disease, uremic cardiomyopathy, NLRP3 inflammasome, apoptosis

## Abstract

Chronic inflammation plays a crucial role in the long-term complications in patients with chronic kidney disease (CKD). This study aimed to assess the role of NLR pyrin domain-containing protein (NLRP3) inflammasome in cardiac contractile dysfunctions in CKD. The cardiac contractile function was evaluated and the expression of NLRP3 inflammasome and related cytokines in the heart was assessed in a murine sham-operated and 5/6 nephrectomy CKD model *in vivo*. *In vitro*, H9c2 cells were treated with uremic toxin indoxyl sulfate (IS), with or without NLRP3 inflammasome inhibition, which was achieved by using small interfering RNA (siRNA)-mediated knockdown of the *NLRP3* gene. Moreover, the activation of nuclear factor κB (NF-κB) signaling and apoptosis marker levels were assessed in the IS-treated H9c2 cells. The results demonstrated that CKD can lead to the development of cardiac contractile dysfunction *in vivo* associated with the upregulation of NLRP3 inflammasome, IL-1β, IL-18, and contribute to the myocardial apoptosis. *In vitro* experiments showed the upregulation of inflammasome, IL-1β, and IL-18 levels, and cell apoptosis in the IS-treated H9c2 cells through the activation of NF-κB signaling pathway. The transfection of cells with si-NLRP3 was shown to alleviate IL-1β, IL-18, and cell apoptosis. Moreover, decreased cell viability induced by IS was shown to be attenuated by IL-1β or IL-18-neutralizing antibody. In summary, CKD can result in the development of cardiac contractile dysfunction associated with the upregulation of NLRP3 inflammasome/IL-1β/IL-18 axis induced by the uremic toxins.

## INTRODUCTION

Inflammasomes are multi-protein platforms that interact with various immune and cellular death pathways [1, 2]. Among these, the best known is the NLR (nucleotide-binding domain leucine-rich repeat-containing receptor) pyrin domain-containing protein 3 (NLRP3) inflammasome, a crucial factor of innate immunity with a role in many diseases, including microbial infections, rheumatic diseases, diabetes, and atherosclerosis [3, 4]. The NLRP3 inflammasome is a protein complex that mediates the activation of caspase-1, interleukin-1β (IL-1β), and interleukin-18 (IL-18), generating a feedback mechanism that perpetuates the inflammatory response [3, 5, 6]. Caspase-1 can be activated within the inflammasome through the binding with the adaptor protein ASC (apoptosis-associated speck-like protein containing a carboxy-terminal CARD), bridging NLRs and caspase-1 [7]. The release of proinflammatory cytokines pro-IL-1β and pro-IL-18 is a complex process involving proteolytic cleavage by caspase-1, which activates IL-1β and IL-18 [8]. IL-1β and IL-18 have been shown to play a role in a variety of cellular processes, including cell apoptosis [9, 10].

NLRP3 inflammasome was recently shown to be present not only in the immune cells, but in the non-immune cells as well, including myocytes and fibroblasts [11, 12]. Increased cardiomyocyte apoptosis may lead to cardiac fibrosis, which plays an important role in cardiac remodeling and results in contractile dysfunction or sudden death [13]. Bracey *et al.* [14] showed that a persistent sterile inflammation and NLRP3 inflammasome presence play a central role in the development of structural cardiomyopathy and heart failure mediated by IL-1β. NLRP3 may be involved in the chronic inflammatory state observed in CKD patients undergoing hemodialysis [15]. However, the effects and mechanisms underlying the inflammasome-related cytokine regulation of apoptosis in uremic cardiomyopathy remain unknown.

In this study, cardiac contractile function was evaluated by echocardiography and the expression of NLRP3 inflammasome and related cytokines in heart were assessed by western blotting in a murine sham-operated and 5/6 nephrectomy CKD model *in vivo*. *In vitro*, the pathogenic role of NLRP3 inflammasome was investigated by treating H9c2 cells with indoxyl sulfate (IS) with or without NLRP3 inflammasome inhibition, which was achieved by using small interfering RNA (siRNA)-mediated knockdown of the *NLRP3* gene. To investigate the mechanism of uremic cardiomyopathy induction by the NLRP3 inflammasome, the activation of nuclear factor κB (NF-κB) signaling and apoptosis marker levels were assessed in the IS-treated H9c2 cells. The findings described in this study may provide insights into the molecular basis of uremic cardiomyopathy as well as potential targets for the treatment of contractile dysfunction.

## RESULTS

### NLRP3 inflammasome-related protein levels were significantly elevated in sera and ventricular tissues of CKD mice

**Figure 1:**
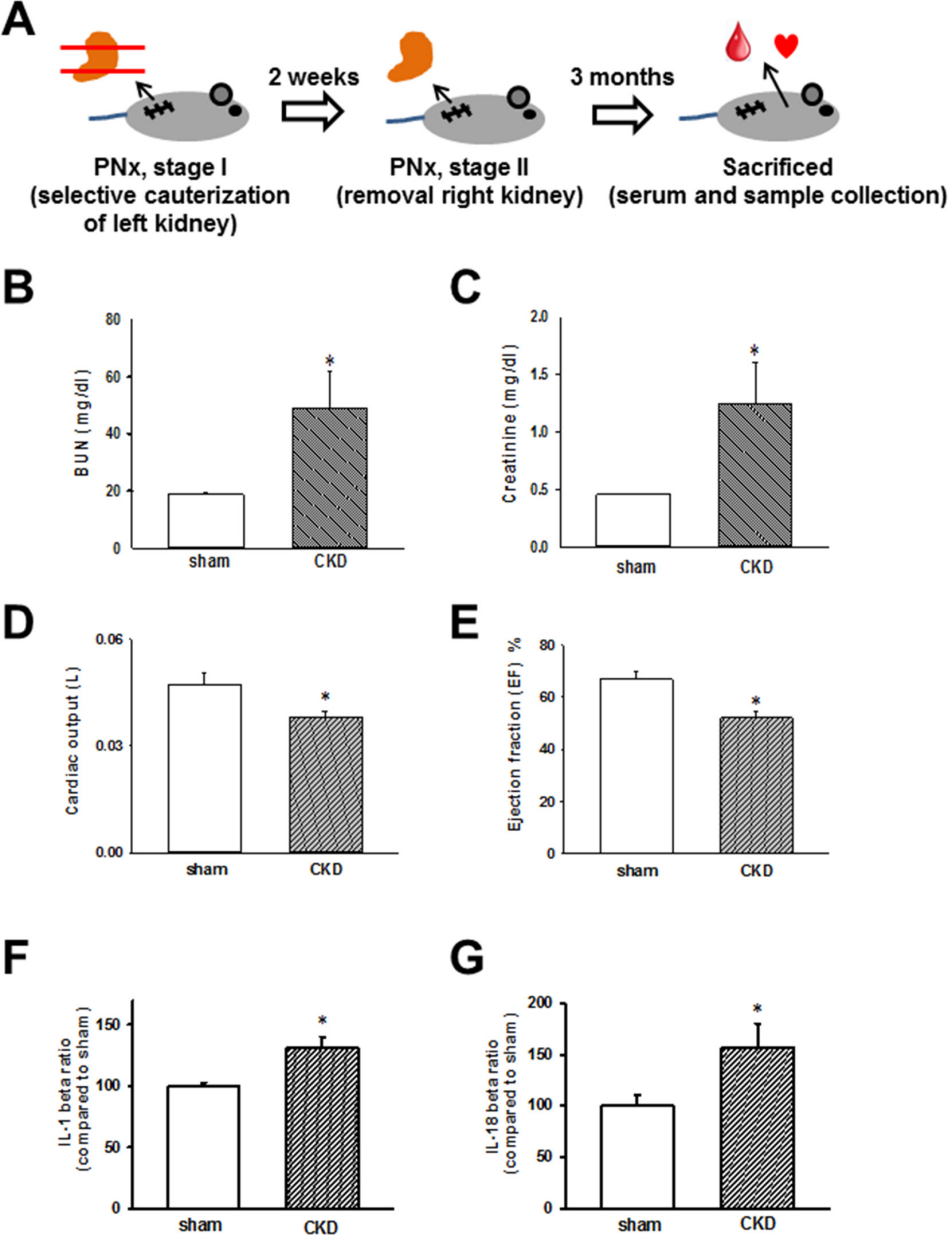
Chronic kidney disease (CKD) effects on heart, biochemical values, and IL-1β and IL-18 concentrations in mouse sera (**A**) Animal experiment schedule. (**B**, **C**) Serum blood urea nitrogen (BUN) and creatinine levels in sham and CKD-operated mice. (**D**, **E**) Cardiac output and ejection fraction (EF) in sham and CKD mice. (**F**, **G**) IL-1β and IL-18 levels in the sera of CKD mice. Values are expressed as mean ± SEM. *p < 0.05, compared with the sham-operated group.

The schedule of the animal experiments is presented in Figure 1A. Serum biochemical data obtained 3 months after nephrectomy are shown in Figure 1B and 1C. The successful induction of experimental renal failure was confirmed by significantly elevated serum blood urea nitrogen (BUN) and creatinine levels in CKD mice. Moreover, echocardiography was used to evaluate and compare cardiac contractile functions in sham-operated and CKD mice. As shown in Figure 1D and 1E, cardiac output and calculated EF were shown to be significantly impaired in CKD mice compared with those in the sham-operated group. As shown in Figure 1F and 1G, IL-1β and IL-18 levels were significantly elevated in the sera of CKD mice.

**Figure 2:**
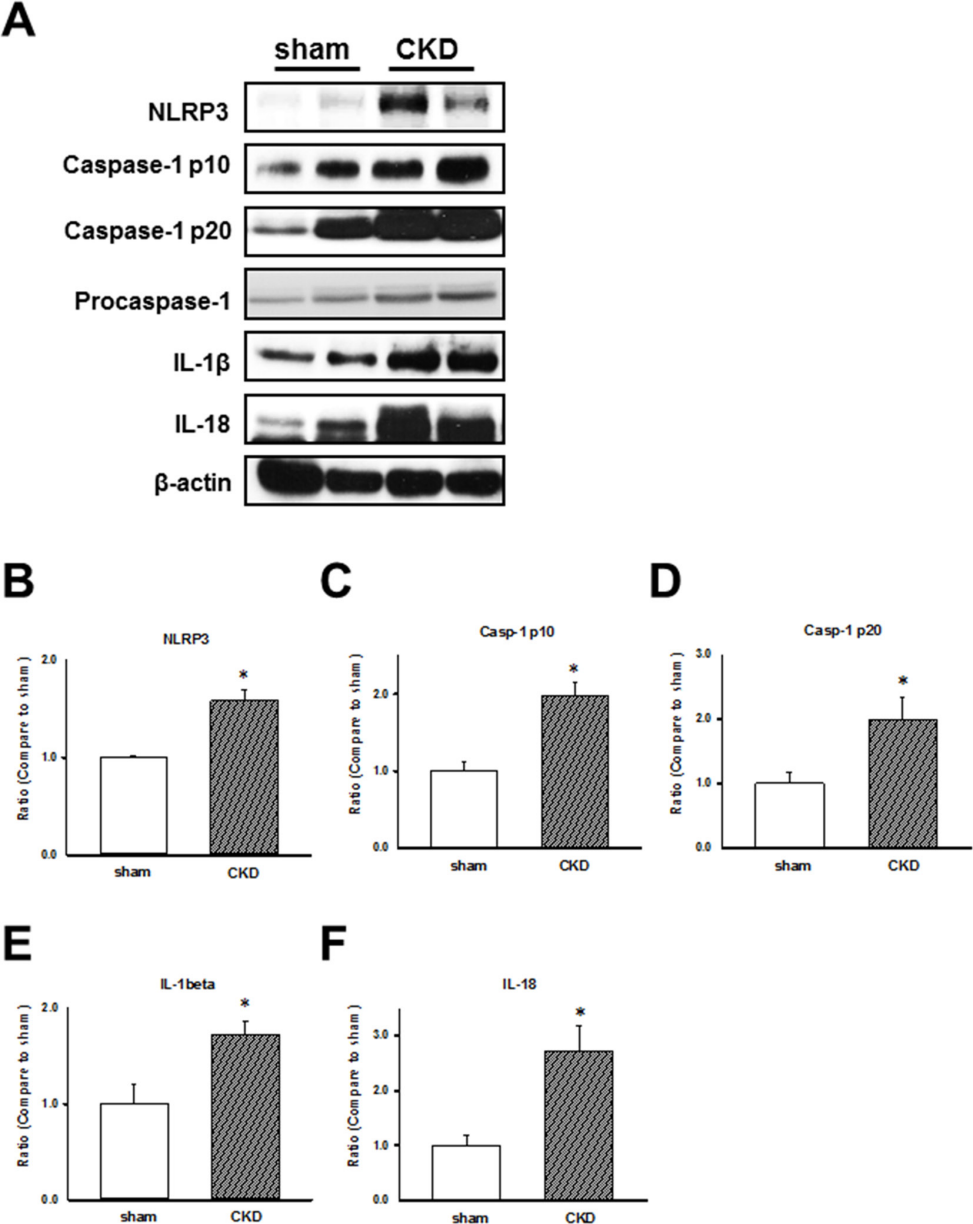
Chronic kidney disease (CKD) effects on inflammasome regulation in mouse ventricular tissues (**A**) Representative immunoblots showing the expression of inflammasome-related proteins in CKD mouse heart tissue. (**B**–**F**) NLRP3, caspase-1 p10, caspase-1 p20, IL-1β, and IL-18 expression levels, normalized to caspase-1 and β-actin expression levels. The levels are expressed as a relative ratio compared with the corresponding control. Values are expressed as mean ± SEM. *p < 0.05, compared with the sham group.

To examine CKD effects on the inflammasome regulation in the cardiac LV tissues of sham-operated and CKD mice, we analyzed the expression of NLRP3 inflammasome and related proteins, caspase-1 p10, p20, and procaspase-1. the results showed that the NLRP3 inflammasome-related protein levels were increased in hearts of CKD mice (Figure 2). We showed that the impaired cardiac contractile function is associated with the upregulation of NLRP3 inflammasome, IL-1β, and IL-18 in CKD mice.

### NLRP3 inflammasome-related protein levels were significantly increased in H9c2 cells

**Figure 3:**
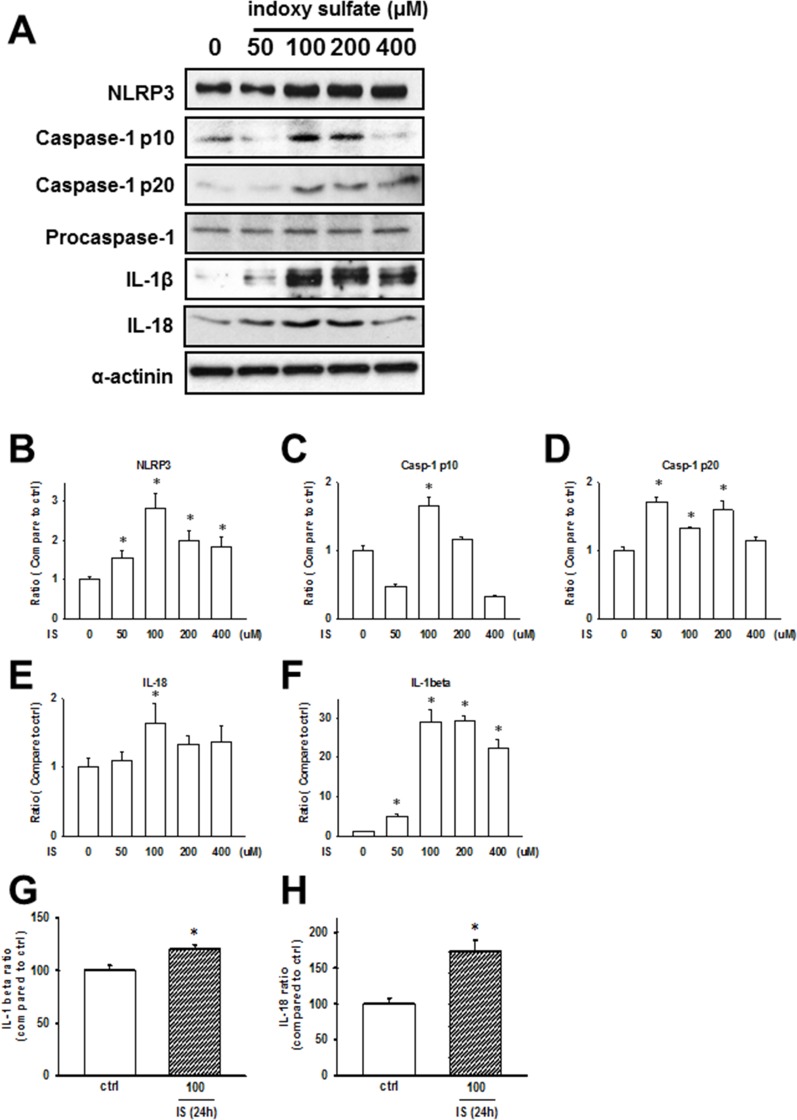
Uremic toxin effects on the inflammasome regulation in H9c2 cells (**A**) Inflammasome-related protein expression in the indoxyl sulfate (IS)-treated cells. (**B**–**F**) Western blot results quantification. Protein levels were normalized to that of α-actinin. (**G**, **H**) IL-1β and IL-18 protein levels in H9c2 cells treated with IS (100 μM) for 24 h. Data are presented as mean ± SEM of three independent experiments. *p < 0.05, compared with the sham group.

IS, a circulating uremic toxin, plays a role in the progression of CKD and can induce UCM *in vivo* and *in vitro* [16–18]. Rat cardiomyocytes, H9c2, were used for studying cellular and molecular mechanisms underlying cardiomyopathy. We treated H9c2 cells with IS (50–400 µM) for 24 h, and the expressions of NLRP3 inflammasome in H9c2 cells increased (Figure 3A–3F). Additionally, secreted IL-1β and IL-18 levels were significantly increased in IS-treated H9c2 samples (Figure 3G and 3H), consistent with the *in vivo* findings.

### Uremic toxin induces NLRP3 inflammasome-related protein expression through NF-κB signaling pathway in H9c2 cells

**Figure 4:**
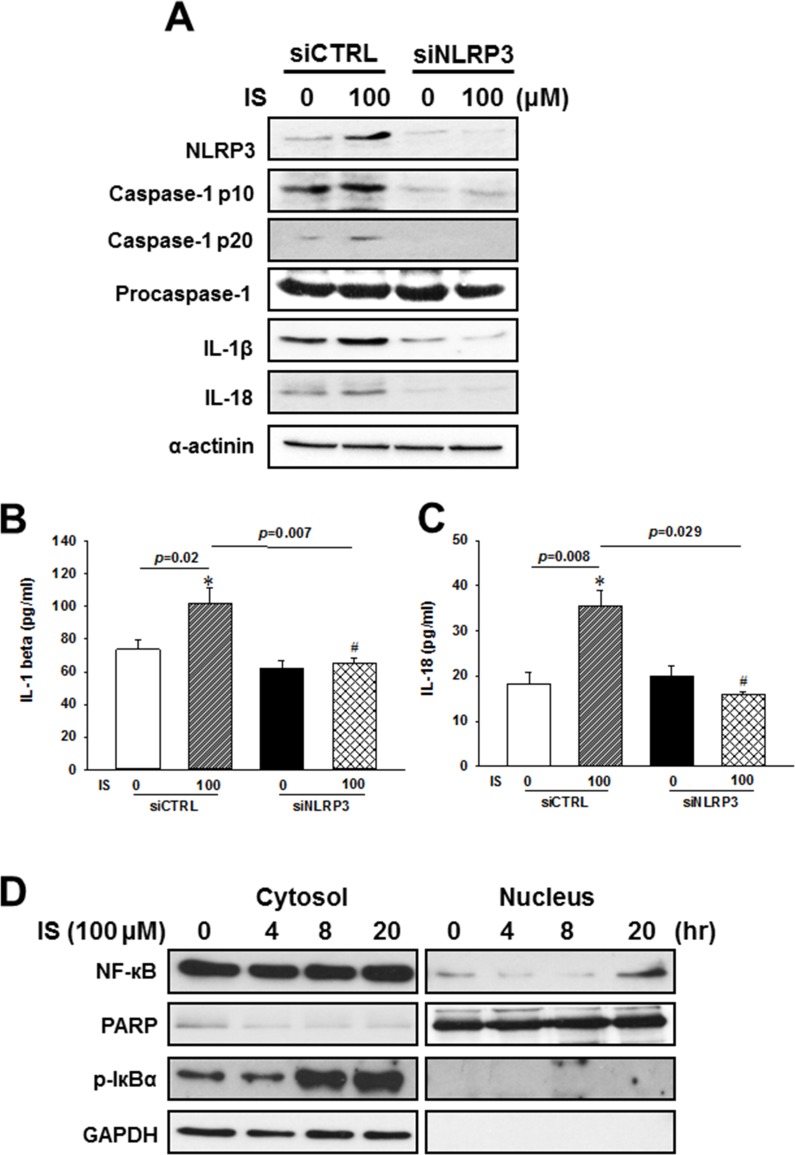
NLRP3 knockdown effects on the regulation of inflammasome-related protein expression in the indoxyl sulfate (IS)-treated H9c2 cells (**A**) Western blot analysis of inflammasome-related protein expression in H9c2 cells treated with NLRP3 siRNA and 100 µM IS. (**B**, **C**) Culture medium was collected to determine the IL-1β and IL-18 levels, and apoptosis-related marker expression in NLRP3 knockdown H9c2 cells. (**D**) NF-кB and Iкbα phosphorylation levels in IS-treated H9c2 cells. The data are presented as mean ± SEM of three independent experiments. *p < 0.05, compared with the control. ^#^*p* < 0.05, compared with the IS treatment group.

To investigate whether NLRP3 is involved in the IS-induced maturation and secretion of IL-1β and Il-18, we used specific siRNAs against NLRP3 in the following experiments. By using NLRP3 siRNAs, we knocked down NLRP3 expression, and inhibited the IS-induced NLRP3 inflammasome protein expressions (Figure 4A) and IL-1β or IL-18 secretions (Figure 4B and 4C) in the H9c2 cells.

NF-κB, a key activator of inflammation, is essential for the expression of NLRP3 inflammasome, pro-IL-1β, and pro-IL-18 [1, 19–21]. Inhibitor of kappa B (IκBα) inactivates NF-κB transcription factor by keeping it sequestered in an inactive state in the cytoplasm [22]. To elucidate the mechanism underlying the uremic toxin-mediated regulation of inflammasome, we investigated NF-κB signaling in H9c2 cells treated with IS. IS treatment was shown to enhance cytosolic Iκbα phosphorylation, and increased the rate of NF-κB translocation into the nucleus of H9c2 cells (Figure 4D).

### Early apoptotic protein expression is induced in the cardiac tissues of CKD mice

**Figure 5:**
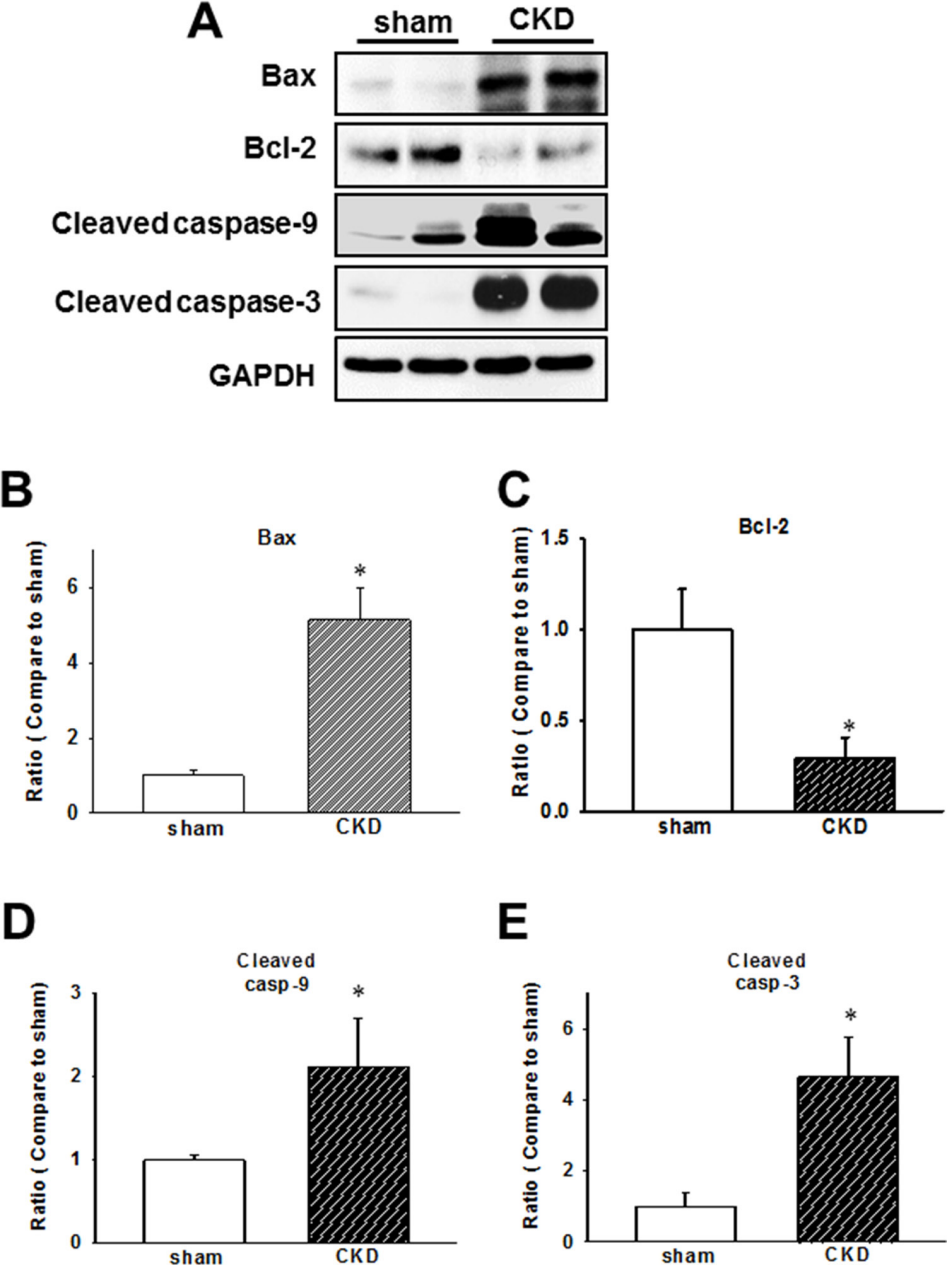
Chronic kidney disease (CKD) effects on the expression of early apoptotic proteins in mouse cardiac tissues (**A**) Expression of early apoptotic proteins in the heart of CKD mice. (**B**–**E**) Quantification of protein levels by using computer-assisted densitometry, and Gapdh levels for normalization. Data are expressed as percentages relative to the corresponding sham control. Values are expressed as mean ± SEM. *p < 0.05, compared with the sham group.

Mitochondria-mediated apoptotic signaling involving the members of Bcl-2 family is well-characterized, while the caspase cascade is believed to be crucial in the regulation of apoptotic processes in cardiomyopathy [23, 24]. In our CKD model, we demonstrated that Bax/Bcl-2 ratio, cleaved caspase-9, and cleaved caspase-3 expression levels are elevated in ventricular tissues (Figure 5).

### IL-1β and IL-18 neutralizing antibodies abrogated the effects of uremic toxin on early apoptosis rate and cell viability in H9c2 cells

**Figure 6:**
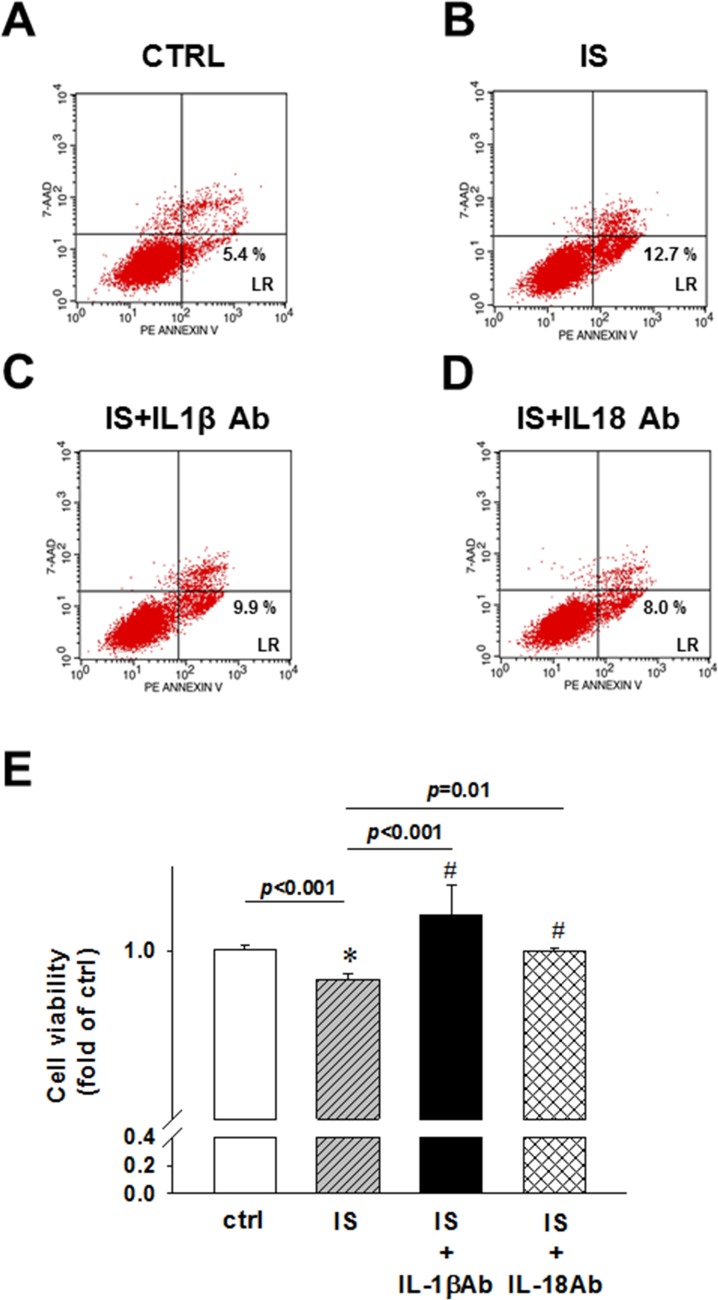
IL-1β and IL-18 neutralizing antibody effects on uremic toxin IS-induced early apoptosis and cell viability of H9c2 cells (**A**) Control. (**B**) Indoxyl sulfate (IS)-treated group. (**C**) IS- and IL-1β antibody-treated group. (**D**) IS- and IL-18 antibody-treated group. H9c2 cells were treated and stained for flow cytometry analysis. Lower right, early apoptosis rate of H9c2 cells. (**E**) MTT assay, showing the viability of IL-1β and IL-18 antibody-treated cells. All data are presented as mean ± SEM. ns=no significance.

IS was shown to induce the apoptosis of kidney mesangial cells [25], whereas the inflammasome-related proteins induce cell apoptosis *in vitro* [26, 27]. Controversially, IL-18 inhibits neutrophil apoptosis through PI3K/ERK signal pathway [10]. To determine the effects of uremic toxin on apoptosis rate, we used IL-1β and IL-18 neutralizing antibodies, and demonstrated that the IS treatment induces early apoptosis, and not necrosis, in H9c2 cells (Figure 6A and 6B). The treatment with IL-1β or IL-18-neutralizing antibodies led to a significant inhibition of apoptosis in H9c2 cells (Figure 6C and 6D). MTT assay results showed that the detrimental effect of IS treatment was attenuated by the addition of IL-1β or IL-18 neutralizing antibody (Figure 6E).

### Uremic toxin was shown to induce apoptosis through NLRP3 activity modulation

**Figure 7:**
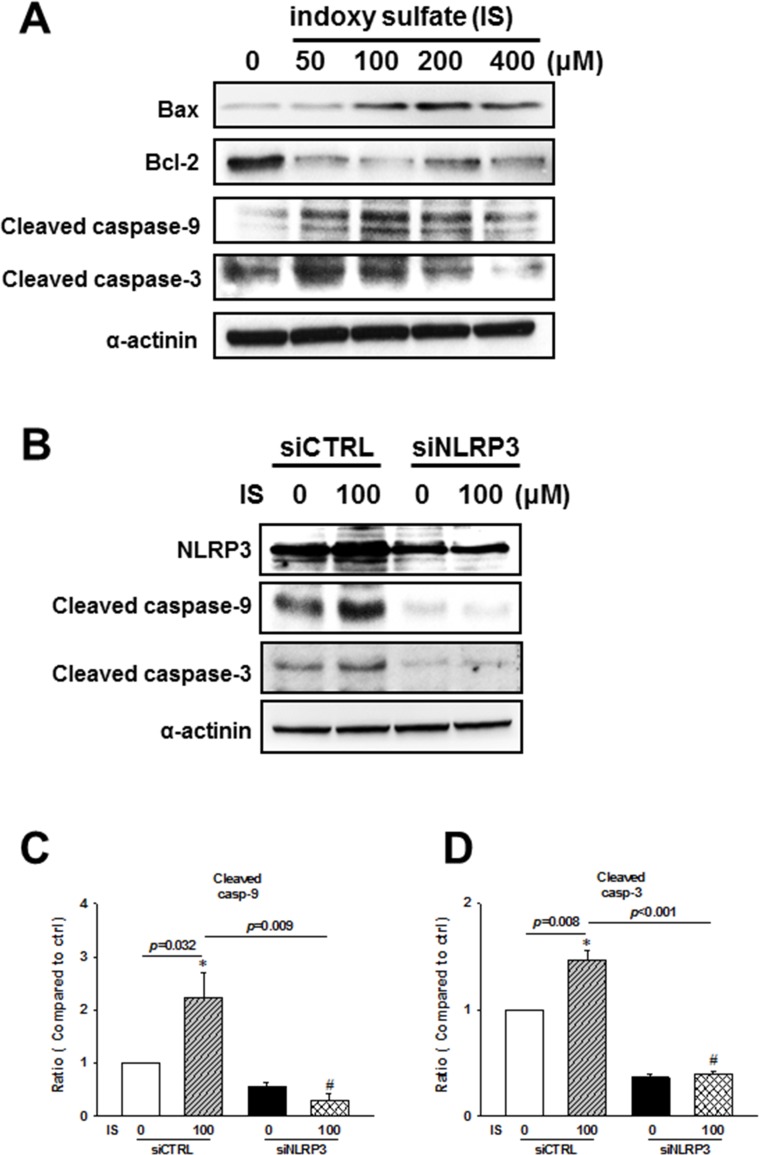
NLRP3 knockdown effects on the apoptosis-related protein expression in indoxyl sulfate (IS)-treated H9c2 cells (**A**) Expression levels of early apoptosis-related proteins in the IS-treated cells. (**B**–**D**) H9c2 cells were treated with NLRP3 siRNA or scrambled siRNA for 24 h, and then stimulated with IS for 48 h, and cleaved caspase-9 and caspase-3 levels were determined in H9c2 cells. Data are presented as mean ± SEM of three independent experiments. *p < 0.05, compared with the control group. ^#^*p* < 0.05, compared with the IS treatment group.

To investigate the molecular mechanism involved in the regulation of IS-induced H9c2 apoptosis, we performed western blot analysis of mitochondria-related apoptotic signaling. IS was shown to induce an increase in Bax/Bcl-2 ratio, cleaved caspase-9, and cleaved caspase-3 expressions (Figure 7A). Additionally, *NLRP3* knockdown inhibited IS-induced expression of cleaved caspase-9 and caspase-3 (Figure 7B–7D).

## DISCUSSION

*In vivo*, PNx-induced CKD lead to the cardiac contractile dysfunction associated with the upregulation of NLRP3 inflammasome, IL-1β, and IL-18 levels, and contribute to myocardial apoptosis. *In vitro*, the upregulation of these molecules and increased cell apoptosis were observed in the IS-treated H9c2 cells, which was shown to be NF-κB-mediated. *NLRP3* suppression was shown to decrease the expression of IL-1β, IL-18, and cell apoptotic markers. Moreover, an IS-induced decrease in cell viability was shown to be attenuated by IL-1β or IL-18 neutralizing antibody. The inhibition of NLRP3 inflammasome-related IL-1β and IL-18 expression may reduce myocardial apoptosis, potentially preserve cardiac contractile function, and decrease uremic cardiomyopathy induced by uremic toxins.

Chronic kidney disease (CKD) represents a strong and independent risk factor for cardiovascular disease (CVD). Among the potential cardiovascular complications, heart failure is the leading one and its prevalence increases with a decrease in kidney function [28]. Compared with that of the non-CKD patients with similar blood pressure levels, CKD patients have a larger left ventricular mass, suggesting the existence of specific CKD-associated factors that contribute to cardiac remodeling [29]. A fundamental response of cardiomyocytes and left ventricular wall to intrinsic and biochemical stress in CKD is hypertrophy, which at first stages may be compensatory, but it inevitably progresses to dilation and cardiac contractile dysfunction. Structural and functional abnormalities may occur at the earliest stages of CKD, without an obvious CVD [30]. The cardiomyopathy associated with CKD is commonly caused by the immune system activation, and the inflammation is considered a non-traditional (CKD-specific) risk factor contributing to CVD development [31, 32].

Inflammasome-related protein activation is required for the maturation and secretion of IL-1β and IL-18 [8]. NLRP3 inflammasome with pro-caspase-1 promotes the secretion of IL-1β and IL-18 [33, 34]. Activation of mitogen-activated protein kinase (MAPK) and NF-κB signaling pathways is crucial for IL-1 activity, including the production and/or release of nitric oxide (NO), cyclooxygenase-2 (COX-2), and other pro-inflammatory mediators [35, 36]. IL-1α and IL-1β are two biologically active isoforms of IL-1, produced as 31 kDa precursors that are stored within the cytosol. Pro-IL-1α is constitutively expressed, whereas pro-IL-1β is transcribed in response to an inflammatory or infectious stimulus [35]. Caspase-1 processes intracellular pro-IL-1β and is produced from the constitutively expressed 45-kDa cytoplasmic pro-enzyme, pro-caspase-1, which requires post-translational processing to form 20- and 10-kDa active forms of caspase-1 [37], following the assembly of the NLRP3 inflammasome. Low active caspase-1, IL-1β, and IL-18 levels induce cytokine production, but higher levels of these molecules can induce cell death or pyroptosis [38].

IL-18 is a key mediator in the host response to infection and the inflammatory response [35, 39]. It is constitutively expressed as a precursor, pro-IL 18 [40], cleaved by either caspase-1 or proteinase-3 into its active form, and released into the extracellular space together with mature IL-1β. Growing evidence suggests that the inflammasome and the IL-1β/ IL-18 axis play an integral part in the pathogenesis of many acute and chronic conditions [41]. Here, 5/6 PNx was shown to induce IL-1β and IL-18 expression in CKD mice and IS-treated H9c2 cells. Furthermore, the expression of NLRP3 inflammasome-related proteins and apoptotic markers in CKD mouse hearts and the IS-treated H9c2 cells was shown to be upregulated. *NLRP3* siRNA knockdown had an opposite effect on the cleaved caspase-9 and caspase-3 expression levels, indicating that IL-1β and IL-18 may bind to the receptors in an autocrine/paracrine manner and induce H9c2 cell apoptosis. These data suggest that the induction of proinflammatory cytokine expression by uremic toxins in CKD can lead to a persistent inflammation in myocardium and myocardial apoptosis, which contributes to uremic cardiomyopathy and cardiac contractile dysfunction development. Cardiomyocyte apoptosis leads to the loss of myocardial contractile unit and finally results in cardiac contractile dysfunction in uremic cardiomyopathy. A decrease in cell viability of the IS-treated H9c2 cells was shown to be attenuated by anti-IL-1β or -IL-18 antibodies. Inflammasome and the IL-1β/IL-18 axis may represent therapeutic targets for the treatment of CKD-related cardiac contractile dysfunction. However, the mechanisms involved in the protection against CKD-induced apoptosis by inflammasome inhibition should be further elucidated.

NLRP3 inflammasome is not limited by traditional microbial stimuli of innate immunity and its connection with autophagy, fibrosis, and pro-inflammatory cytokines has broader implications for a variety of kidney disease [42]. This sterile renal inflammation is considered a central therapeutic target for the reduction of chronic renal injury and decline in CKD progression [43]. Persistent inflammation leads to the progressive renal fibrosis and loss of renal function, together with the induction of other CKD-related complications, such as cardiovascular disorders [44–46]. The NLRP3 inflammasome is activated in the ischemic heart and the inhibition of the NLRP3-initiated inflammasome during experimental myocardial infarction minimizes the activation of caspase-1 in the heart and improves LV remodeling and function [14, 47–50]. Moreover, NLRP3 inflammasome is also involved in the development of myocardial dysfunction with sepsis and diabetes in animal models [11, 12]. We showed that the uremic toxin-related NLRP3 inflammasome upregulation may be extrapolated to uremic cardiomyopathy and cardiac contractile dysfunction.

IS, a tryptophan metabolite, is a representative uremic toxin, which may initiate cellular responses, phagocytosis, and particularly apoptosis [51]. The accumulation of uremic toxins impairs hemostasis and increases the rate of infections and malignancy due to its effects on the immune system [52]. In uremic patients, IS serum levels are markedly increased, which induces oxidative stress [53]. Oxidative stress and chronic inflammation are strongly related and contribute to long-term complications in HD patients, [54, 55]. In this study, CKD induced the upregulation of inflammasome-related protein expressions and apoptotic signaling pathway members, activating mitochondria-associated apoptotic signaling, through the modulation of Bcl-2 family members and caspase cascade in CKD mouse hearts and IS-treated H9c2 cells. Uremic toxin was shown to regulate inflammasome-related protein expressions through the activation of NF-κB-mediated signaling in H9c2 cells. Our results indicate the crucial role of IS in the regulation of NLRP3 inflammasome in uremic cardiomyopathy.

There are some limitations in this study. First, although we demonstrated IL-1β and IL-18 may bind to the receptors in an autocrine/paracrine manner and induce H9c2 cell apoptosis, cardiomyocytes are not the only source of IL-1β and IL-18. The effect of IS on other immune cells should also be response for the elevation of IL-1β and IL-18 in serum. Second, there are multiple signaling pathways involved in the detrimental effects of IS and therefore lead to uremic cardiomyopathy and IS in not the only uremic toxin in CKD. Third, cardiac contractile dysfunction is a complex disease entity in patients with CKD. Several CKD-specific factors, including abnormal calcium and phosphate metabolism, uremic toxins, anemia, inadequate dialysis, and increased oxidative stress, have been shown to contribute to the development of uremic cardiomyopathy. Although we clearly demonstrated the role of NLRP3 inflammasome in the development of cardiac contractile dysfunction, more extensive *in vivo* and *in vitro* studies are necessary to investigate the complexity of this disease entity.

**Figure 8:**
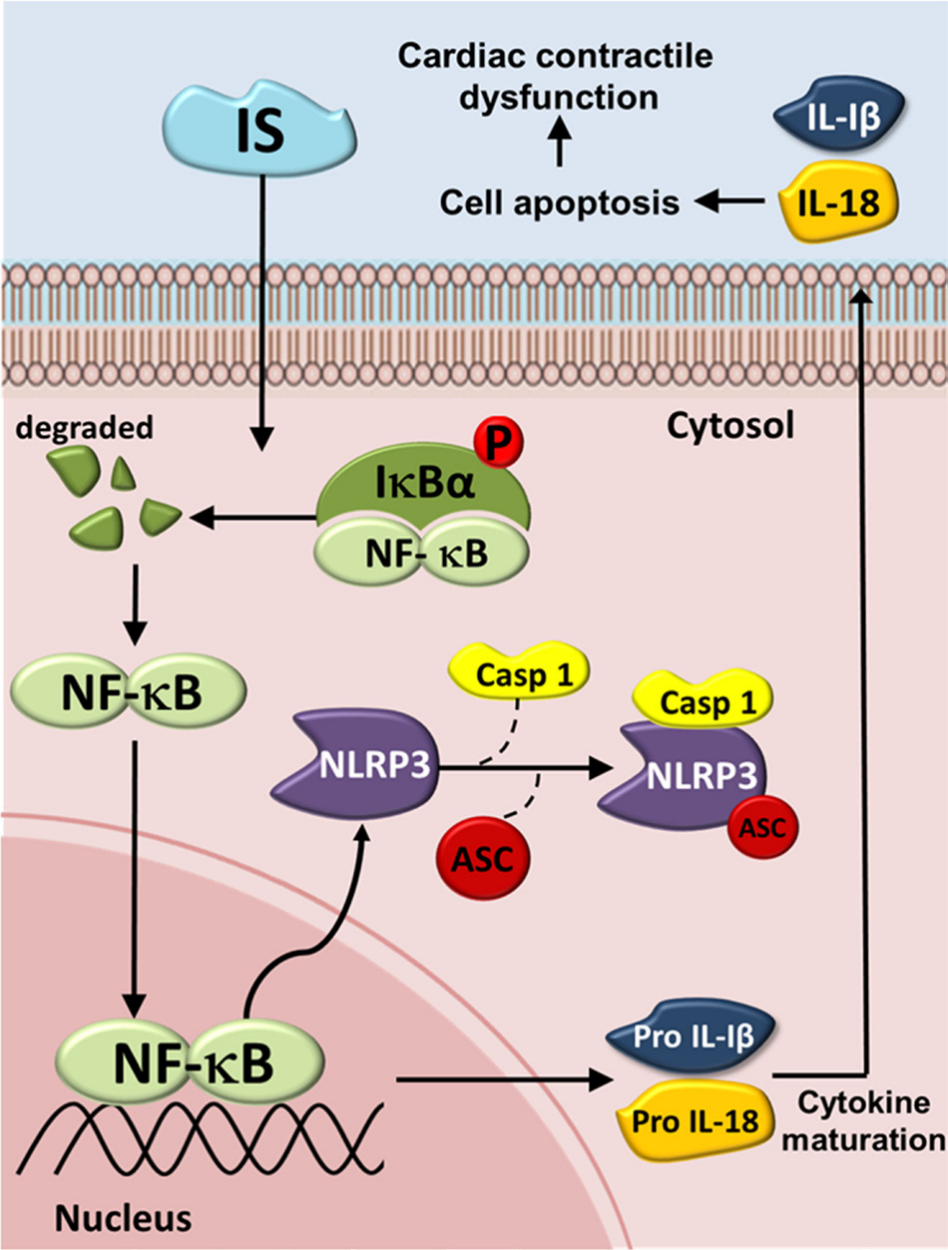
Schematic diagram of molecules mediating indoxyl sulfate (IS) effects on inflammasome in UCM The IS-induced increase in the expression of IL-1β and IL-18 may be mediated through the NLRP3/caspase-1 signaling pathway, and the secreted IL-1β and IL-18 may bind to their receptors, inducing cell apoptosis by activating the early apoptotic cascade. IS = Indoxyl sulfate; Iκbα = inhibitor of kappa B; NF-кB = Nuclear factor kappa B; NLRP3 = NLR pyrin domain-containing protein 3; ASC = Apoptosis-associated speck-like protein; Casp1 = Caspase-1; IL-1β = Interleukin-1β; IL-18 = Interleukin-18.

In conclusion, The NLRP3 inflammasome is increasingly being recognized as an integral component of the pathogenesis of many renal diseases and their complications. Taken together, we here show a possible mechanisms underlying the IS-induced inflammasome expression and cardiomyocyte apoptosis (Figure 8). IS treatment was shown to increase the expression of inflammasome by inducing cytosolic Iκbα phosphorylation and NF-κB nuclear translocation, which may lead to a subsequent binding of this molecule to the *NLRP3* promoter, further inducing IL-1β and IL-18 expression and secretion. This may, subsequently, contribute to H9c2 cell apoptosis. The results of this study may help develop a novel strategy for the treatment of patients with UCM. Inflammasome may represent a promising therapeutic target for the treatment of CKD-related cardiomyopathy.

## MATERIALS AND METHODS

### Animal experiments

CKD cardiomyopathy was induced *in vivo* using partial nephrectomy (PNx) at a C57BL/6J background as described previously [56, 57]. Animal experiments were approved by the local animal care committee of National Defense Medical Center (IACUC-16-266). Animals were housed in a light- and temperature-controlled room with free access to deionized drinking water and standard chow consisting of 0.28% (wt/wt) NaCl, 1.00% (wt/wt) Ca, 0.22% (wt/wt) Mg (LabDiet, Richmond, IN, USA). After the acclimatization, mice were randomly assigned to two groups: sham-operated and PNx mice. PNx was performed on 4-month-old male C57BL/6 mice by selective cauterization of the entire upper and lower poles of the left kidney via a Bovie high-temperature fine-tip cautery (Aaron Medical, St. Petersburg, FL, USA), leaving an intact 2-mm segment around the hilum (PNx, stage I). This procedure was followed by the removal of the right kidney after 2 weeks (PNx, stage II). The animals were euthanized 3 months after sham operation or PNx by CO_2_ inhalation. Blood was collected and the hearts were dissected and processed for further analyses.

### Echocardiography

Echocardiography was performed using a Philips iE33 ultrasound imaging system (Philips Medical Systems, Best, Netherlands) equipped with a 7-15 MHz linear array transducer. Anesthesia was performed using 3% isoflurane inhalation, until animals were sedated, and then it was maintained with 1% isoflurane during the echocardiographic examination. After obtaining two-dimensional left ventricular () short-axis images, M-mode traces were acquired for the measurement of LV wall thickness and chamber dimensions at diastole and systole, echocardiography-derived ejection fraction (EF). All measurements were averaged for five consecutive cardiac cycles and were performed in a blinded manner by an experienced technician (Y-H Chen).

### Cell culture and treatment

The H9c2 rat myoblast cell line (BCRC60096) was purchased from the Bioresource Collection and Research Center of the Food Industry Research and Development Institute (Taipei, Taiwan). Cells were grown in Dulbecco’s Modified Eagle’s Medium (DMEM) supplemented with 10% fetal bovine serum (FBS), 150 U/mL penicillin, and 150 mg/mL streptomycin. Cells were incubated at 37°C in 5% CO_2_/95% air. Confluent cells were detached using a 0.05% trypsin/0.02% EDTA solution and subcultured to the second passage in six-well culture plates. H9c2 cells were treated with IS (50–400 μM) in the presence or absence of IL-1β or IL-18 neutralizing antibody (1 μg/mL).

### IL-1β and IL-18 concentration measurements using enzyme-linked immunosorbent assay (ELISA)

IL-1β (Koma Biotech Inc., Seoul, Korea) and IL-18 (MBL Int., Woburn, MA, USA) concentrations in the mouse sera or H9c2 conditioned media were determined using the ELISA kits according to the manufacturers’ instructions. Cell culture media were collected and centrifuged at 3000 rpm for 1 min at 4°C. The supernatants were collected, and the concentrations of IL-1β and IL-18 were determined.

### Nuclear extraction

To examine the effect of CKD on nuclear translocation of NF-κB (p65), the NE-PER nuclear and cytoplasmic extraction reagents (Thermo Fisher Scientific, Rockford, IL, USA) were used and the extraction was performed according to the manufacturer’s protocol. Briefly, cell pellets were resuspended in the cytoplasmic extraction reagent I buffer containing protease inhibitor cocktail (Sigma-Aldrich), and then the cells were lysed in the cytoplasmic extraction reagent II buffer, to release cytoplasmic proteins. After the centrifugation at 16,000 × *g* for 5 min at 4°C, the insoluble pellet, containing nuclear proteins, was washed with ice-cold phosphate-buffered saline (PBS) two times and then incubated in nuclear extraction reagent buffer containing protease inhibitor cocktail (Sigma-Aldrich).

### NLRP3 knockdown by siRNA

Cells were transfected with scrambled control siRNA (cat. D-001810-10, Dharmacon, Lafayette, CO, USA) or NLRP3-pooled specific siRNA (final concentration, 25 nM; cat. L-084509-02-0005, Dharmacon, Lafayette, CO, USA) by using INTERFERin transfection reagent (PolyPlus Transfection, Illkirch, France) according to manufacturer’s instructions.

### MTT assay

The MTT (3-(4,5-dimethylthiazol-2-yl)-2,5-diphenyltetrazolium bromide) tetrazolium reduction assay is based on the mitochondrial activity, which leads to the production of water-insoluble formazan salt. Cells were seeded in 24-well plates and treated. Afterward, 10 μL MTT (final concentration, 0.5 mg/mL) were added to each well, followed by the incubation for 4 h at 37°C. Culture medium was removed by aspiration, and the cells were washed twice with PBS, after which 100 μL of dimethyl sulfoxide (DMSO) were added to dissolve blue formazan in viable cells. Absorbance was read at 570 nm using a microplate reader (Sunrise, Tecan, Germany). Cells incubated with control medium were considered 100% viable. Cell viability percentage = the optical density (OD) value of each treated group/OD value of control group × 100%. The concentration of icariin used in further experiments was selected based on the MTT results.

### Apoptosis and necrosis evaluation using flow cytometry

Apoptotic cell death rate was assessed by using the annexin V-phycoerythrin (PE) apoptosis detection kit (BD Pharmingen, San Diego, CA, USA) according to the manufacturer’s protocol. Briefly, the cells were harvested by trypsinization, centrifuged, and washed twice in cold PBS. Afterwards, they were resuspended in 100 µL of 1× binding buffer at a concentration of 1 × 10^6^ cells/mL. After the addition of 5 µL of annexin V PE and 5 µL of 7-amino-actinomycin (7-AAD), the cells were gently vortexed and incubated for 15 min at room temperature (25°C) in dark. The cell samples were diluted in 400 µL of 1 × binding buffer and analyzed by flow cytometry within 1 h using a Nucleocounter NC-3000 instrument (ChemoMetec Inc, Allerod, Denmark). Compensation and plot quadrants were determined by using the unstained cells and cells stained with either annexin V-PE or 7-AAD alone. The gating strategy was as follows: an initial forward scatter *vs.* side scatter gating, to exclude debris, followed by annexin V-PE and 7-AAD gating, to distinguish the apoptotic from viable cells. By using this gating strategy, annexin V-negative/7-ADD negative cells were defined as viable, annexin-positive/7-AAD negative cells as early apoptotic, annexin V-negative/7-ADD-positive cells as dead cells, and annexin V-positive/7-ADD-positive cells as late apoptotic or necrotic cells.

### Western blot analysis

Tissues were homogenized in lysis buffer (1 M Tris-Cl PH 8.0, 1.2 M NaCl, 0.5% v/v Nonidet P-40) with 10 μL/mL of protease inhibitor cocktail freshly added (Sigma-Aldrich) for 15 min on ice, followed by the centrifugation at 14000 × g for 15 min at 4°C. Protein content was determined in the supernatants, according to the manufacturer’s instructions (Bio-Rad). Samples were boiled for 5 min in reducing sample buffer with 2-mercaptoethanol (5 μL/100 mL) and then resolved using 10% SDS-PAGE. Separated proteins were transferred to nitrocellulose membranes, which were then blocked in 5% milk in PBS with 0.01% Tween-20 (PBST) overnight at 4°C. After blocking, the membranes were incubated in diluted primary antibody solutions for 1 h at room temperature, which was followed by washing in PBST for 15 min, three times. Membranes were then incubated with the diluted (1:10000) secondary horseradish peroxidase (HRP)-conjugated antibodies for 1 h, followed by the washing in PBST for 15 min three times. ECL Plus western blotting detection reagents (Amersham Biosciences) and Kodak X-ray films were used for the visualization of protein bands. Primary antibodies diluted in bovine serum albumin (BSA) were as follows: rabbit anti-NLRP3 (Cell Signaling Technology), rabbit anti-caspase-1 p10 (Santa Cruz Biotechnology, USA), goat anti-caspase-1 p20 (Santa Cruz Biotechnology, USA), rabbit anti-caspase-1 (Proteintech, China), mouse anti-IL-1β (Santa Cruz Biotechnology, USA), mouse anti-IL-18 antibody (Santa Cruz Biotechnology, USA), rabbit anti-Bcl-2 (Proteintech, China), mouse anti-caspase-9 (Cell Signaling Technology, USA), rabbit anti-caspase-3 (Cell Signaling Technology, USA), mouse anti-p-Iкbα (Santa Cruz Biotechnology, USA), rabbit anti-NF-кB (Cell Signaling Technology, USA), rabbit anti-glyceraldehyde-3-phosphate dehydrogenase (GAPDH) (MBL, Japan), and mouse anti-β-actin (Sigma-Aldrich, USA) antibodies. Protein band intensity in each blot was normalized to the levels of Gapdh, and relative protein levels were quantified using ImageJ software.

### Statistical analysis

Data were expressed as mean ± standard error of the mean (SEM). Differences between groups were examined for statistical significance using Kruskal-Wallis test, if appropriate. Statistical significance was set at *p*-values < 0.05.

## Abbreviations

ASC: apoptosis-associated speck-like protein
BUN: blood urea nitrogen
Casp1: Caspase 1
CKD: chronic kidney disease
CVD: cardiovascular disease
EF: ejection fraction
GAPDH: glyceraldehyde-3-phosphate dehydrogenase
IL: interleukin
IL-1β: interleukin-1β
IL-18: interleukin-18
IS: indoxyl sulfate
LV: left ventricle
LVH: left ventricular hypertrophy
MAPK: mitogen-activated protein kinase
MTT: 3- (4,5-dimethylthiazol-2-yl)-2,5-diphenyltetrazolium bromide
NF-κB: nuclear factor κB
NO: nitric oxide
NLR: nucleotide-binding domain leucine-rich repeat-containing receptor
NLRP3: NLR pyrin domain-containing protein 3
PARP: poly ADP-ribose polymerase
PNx: partial nephrectomy
siRNA: short interfering RNA
UCM: uremic cardiomyopathy
